# Environmental Stress Cracking of High-Density Polyethylene Applying Linear Elastic Fracture Mechanics

**DOI:** 10.3390/polym14122415

**Published:** 2022-06-14

**Authors:** Maximilian Thuy, Miquel Pedragosa-Rincón, Ute Niebergall, Harald Oehler, Ingo Alig, Martin Böhning

**Affiliations:** 1 Bundesanstalt für Materialforschung und—Prüfung (BAM), Unter den Eichen 87, 12205 Berlin, Germany; maximilian.thuy@bam.de (M.T.); miquelpedragosar@iqs.url.edu (M.P.-R.); ute.niebergall@bam.de (U.N.); 2 Fraunhofer Institute for Structural Durability and System Reliability LBF, Research Division Plastics, Schlossgartenstraße 6, 64289 Darmstadt, Germany; harald.oehler@lbf.fraunhofer.de (H.O.); ingo.alig@lbf.fraunhofer.de (I.A.)

**Keywords:** crack propagation, environmental stress cracking, fracture toughness, crop protection products, high-density polyethylene, fracture surface structure, stress intensity factor, craze–crack mechanism, linear elastic fracture mechanics

## Abstract

The crack propagation rate of environmental stress cracking was studied on high-density polyethylene compact tension specimens under static loading. Selected environmental liquids are distilled water, 2 wt% aqueous Arkopal N100 solution, and two model liquid mixtures, one based on solvents and one on detergents, representing stress cracking test liquids for commercial crop protection products. The different surface tensions and solubilities, which affect the energetic facilitation of void nucleation and craze development, are studied. Crack growth in surface-active media is strongly accelerated as the solvents induce plasticization, followed by strong blunting significantly retarding both crack initiation and crack propagation. The crack propagation rate for static load as a function of the stress intensity factor within all environments is found to follow the Paris–Erdogan law. Scanning electron micrographs of the fracture surface highlight more pronounced structures with both extensive degrees of plasticization and reduced crack propagation rate, addressing the distinct creep behavior of fibrils. Additionally, the limitations of linear elastic fracture mechanisms for visco-elastic polymers exposed to environmental liquids are discussed.

## 1. Introduction

Crack propagation in high-density polyethylene (PE-HD) materials is of critical importance in the industry of packaging and transportation of dangerous goods. Slow crack growth (SCG) and, in particular, environmental stress cracking (ESC) are complex damage mechanisms that especially affect PE-HD in container and pipe applications and are known to be responsible for premature failure due to cracking in these polyolefin materials. For material developers and packaging or pipe manufacturers, it is therefore of importance to determine the environmental stress cracking resistance (ESCR) of the packaging material. Standardized methods include the full-notch creep test (FNCT) [1,2,3], Pennsylvania edge notch test (PENT) [4,5,6], or cracked round bar test (CRB) [7,8,9]. Within these methods, the time to failure is commonly the characteristic criterion to assess ESCR. In order to shorten testing times or to study specific fracture mechanisms, cyclic loading is often conducted through fatigue testing, as also demonstrated with the CRB test [10,11,12,13]. A different and widely used standardized method to determine a material’s fracture toughness employs displacement controlled experiments of notched specimen [14]. For fracture mechanic experiments under static load within environmental media, such as organic liquids or aqueous surfactant solutions, no standardized methods have been currently defined for evaluating crack propagation of polymeric materials such as PE-HD. Based on a previous study of the damaging effect of crop protection products on PE-HD using FNCT [15], it is the focus of this study to quantify crack propagation in more detail using such fracture mechanics approaches. For application reasons and in order to support standardization, we have chosen two model liquids established for testing of container materials for crop protection products. Besides the surface–active model liquid (PFL-FR 2344) and the solvent-based model liquid (PFL-FR 2323), a standard aqueous surfactant solution (2 wt% aq. Arkopal N100) and distilled water for comparison are used. The compact tension (CT) specimen is selected for this study as it features a simple geometry for monitoring crack propagation rate by use of crack opening measurements. The experiments were performed under controlled static load while exposed to these four environmental media at a slightly elevated temperature.

**Figure 1:**
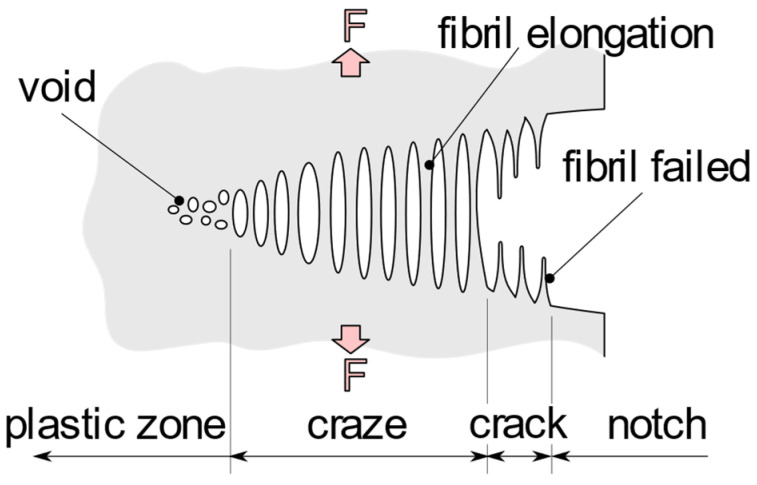
Schematic illustration of the process zone at the notch root or crack tip. Initiation of voids in the plastic zone by stress concentration at the crack tip due to external loading. Voids grow in a plane perpendicular to the direction of stress and form into fibrils that temporarily stabilize the craze zone. As the local stress in the fibrils increases, they fail and the craze–crack transition occurs. Craze–crack propagation right to left (remade after [17,25]).

**Figure 2:**
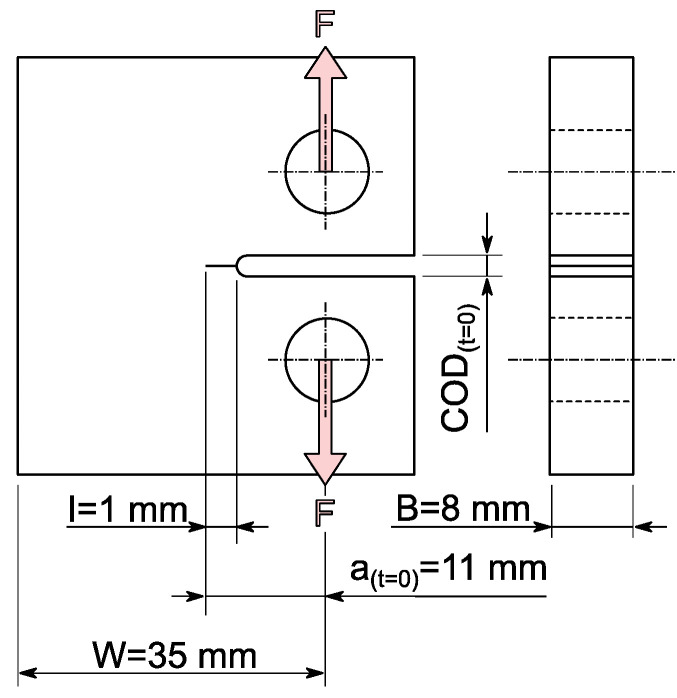
Compact Tension specimen with main dimensions and experimental parameters with I: notch depth by razor blade, W: specimen length, B: specimen thickness, $a_{t=0}$: initial crack length, as well as COD_(*t*=0)_: initial crack opening displacement and F: applied force.

The conditions at the crack tip (Figure 1) can be described by applying linear elastic fracture mechanics (LEFM). This requires linear elastic material behavior at a sharp crack with small-scale plasticity; in relation to this study, the entire specimen is considered as linear elastic so that inelastic processes of a smaller scale can be neglected [12,16,17]. According to the concept of LEFM, the stress distribution at the crack tip and its closer surrounding is described by a single parameter, the stress intensity factor $K_{I}$ (index I refers to the opening mode I: normal to the crack plain). The amount of stress rise depends on crack length a on the sample geometry and on the applied external load. $K_{I}$ can be used to get a measure allowing for comparison of different cracks in conditions where these parameters are different. For the CT specimen, the stress intensity factor is given by:

(1) $$K_{I}=\frac{F}{B\sqrt{W}}\;f\left(\frac{a}{W}\right)$$

where F is the applied force, B is the specimen thickness, and W the specimen length as defined in Figure 2. $f\left(a\;W^{-1}\right)$ is a geometry function of the specimen configuration.

The mechanism of the SCG and ESC requires loads below the yield stress of the material. If, on the other hand, the stress intensity factor exceeds a critical value $K_{Ic}$ (the fracture toughness), instable crack propagation in brittle materials or plastic deformation (e.g., in the case of PE-HD) occurs, which leads to instable or ductile fracture, respectively. However, for stable crack propagation below the yield stress locally at the crack tip, the propagation rate is expected to be a power law function of the applied stress intensity factor [18,19,20], referring to the Paris–Erdogan law [21] for cyclic loading:

(2) $$\frac{da}{dt}=C\cdot K_{I}^{m}$$

with a being the crack length and t the time. C and m are empirical parameters, depending on the material, temperature, and other environmental conditions. The stable crack propagation is found in the central linear region when logarithmic crack propagation velocity is plotted against the logarithmic stress intensity factor. The power law dependence of crack propagation velocities for static load (creep crack growth) as a function of the stress intensity factor is verified for the propagation stage in references [18,19,22,23].

Under low stress, stable crack propagation in PE-HD is generally observed undergoing the craze–crack mechanism of SCG depicted in Figure 1. Due to external loads, stress concentrations occur at notches, scratches, or material imperfections which lead to microscopic void nucleation in amorphous phases between crystallites and crystalline lamellar structures [16]. This nucleation of voids is a result of small-scale plastic flow and orientation of polymer chains [17,18,19] followed by further elongation and cavitation within the amorphous phase. Such cavities grow into crazes in a plane perpendicular to the external stress in front of the stress concentration [20]. These crazes consist of highly oriented fibrils being elongated. The high orientation of the polymer chains in the direction of load maintains the craze to be stabilized by the fibrils. Failure due to breakage or disentanglement of the polymer chains as a result of the locally increasing stress in a fibril initiates the craze–crack mechanism. Although the elastic part of the deformation in the fibrils and membranes reverses after the craze–crack transition, a large part of the deformation is irreversible and plastic. The major remaining elongation in the material is conserved and forms the fracture surface microstructure of the antecedent craze–crack mechanism [15,21]. This mechanism of SCG, without the influence of a liquid environmental medium, yields a similar pattern and may be accelerated to some extent by undergoing ESC if a liquid medium is present [3,24].

A change in surface or interface energy can, for example, be caused by contact with the environmental medium. Wetting of a liquid with a similar surface energy to the polymer material within a crack or voids of the craze leads to a reduction of the energy contribution needed for creation of new interfacial area in connection to the craze–crack mechanism described above. It is well known that the surface energy of liquids can be determined by measuring surface tension. In environmental media with low surface tension, as is the case for aqueous solutions of detergents (amphiphilic structures often forming micelles), test specimens fail faster due to the craze–crack mechanism than they do in media with relatively higher surface tensions [3,24,26]. The energetic consideration of the formation of new surfaces or interfaces between PE-HD and the environmental medium, as they are created during diffuse small-scale yielding in crazing (Figure 1), influences the propagation of cracks [27,28]. The energy required for the creation of new surface area is related to the amount of covalent bonds crossing the created surface and the surface energy, defined by the van der Waals as cohesion energy between molecules. Further details on energy considerations can be found in [25,27,28,29].

Therefore, in this study, test liquids are discussed which accelerate crack growth by reduction of the energy required to form new surfaces: those which are in part sorptive, and plasticize the polymer matrix, and distilled water which is not soluble in the polymer and hardly accelerates crack growth. The novelty is the first appearance of crack–crack growth phenomena in plasticized material, additionally surrounded by the plasticizing liquid (organic solvents) described by the fracture mechanics concept of LEFM. It is important to note that conditions such as temperature, force, and specimen geometry are adjusted sufficiently for crack growth to occur, especially in the case of plasticized PE-HD material. An analysis of the fracture surface, e.g., by scanning electron microscopy, not only indicates the appearance of stable crack propagation (craze–crack mechanism), but also the structural features of the fracture surface which correlate with the stress intensity factor [30,31].

## 2. Materials and Test Liquids

### 2.1. Specimen Preparation

For these studies, a PE-HD type (Lupolen 5261 Z) is chosen, which is typically used for open top drums and jerry cans for packaging of dangerous goods. It is described with properties of good chemical resistance and fair ESCR. The examined material was kindly provided by LyondellBasell (Basell Polyolefine GmbH, Frankfurt am Main, Germany).

To avoid orientation or preferred directions of the polymer microstructure in the later test specimen, it is machined from hot pressed sheets. The powder is pressed at 180 °C for 5 min at a pressure of 10 MPa to form sheets of 8 mm thickness [32,33]. Cooling down slowly at 15 K min^−1^ ensures low residual stresses, which are additionally reduced by subsequent annealing at 100 °C for 3 h. The CT specimens are milled from the pressed sheets, Figure 2. Directly before the experiment a I = 1 mm deep notch is applied using a toggle press and a razor blade (blade tip radius < 10 μm).

### 2.2. Test Liquids

Since the purpose of this study is to classify different environmental media for their effects on crack propagation and fracture behavior, four different test liquids are employed. Distilled water is used as a quasi-neutral environment. Additionally, an aqueous detergent solution known to accelerate environmental stress cracking is considered [3,26,34]. In particular, this study employs a 2 wt% aqueous solution of Arkopal N100, which is often used in other contexts than crop protection products as a standard medium evaluating the ESCR of PE-HD materials [2].

The remaining two test liquids in this study are model liquids consisting of various individual components typically found in crop protection products serving as solvents and emulsifiers for the actual biologically active ingredients. These model liquids, PFL-FR 2323 and PFL-FR 2344 are corresponding mixtures without the actual biological active ingredient, Table 1 and Table 2. The model liquid PFL-FR 2323 contains more than 75 wt% of solvents, supplemented with components possessing emulsifying properties for a stable solution. As shown in a previous study [15], the solvents of this model liquid have a swelling/sorptive effect on the PE-HD. PFL-FR 2344 consists of 60 wt% of a surfactant component and is dissolved in water with sodium chloride. Additionally, a minor amount of one of the components from the group of solvents (1-methoxy-2-propanol) is included. In summary, a solvent-based test liquid and a water-based test liquid were utilized to compare the damage capability of these model liquids for crop protection products with the other environmental test liquids introduced above. PFL-FR 2323 and PFL-FR 2344 actually originate from the approval testing of container materials for dangerous goods in Germany. They serve as strongly ESC-accelerating standardized model liquids in various laboratory tests for the approval of PE-HD materials [35,36,37].

The surface tensions between the test liquids and the gas phase were determined with a tensiometer (K11 MK3 of Krüss GmbH, Hamburg, Germany), performing the plate method according to Wilhelmy at a test temperature of 40 °C, Table 3 [38]. A surface free energy of 34.2 ± 1.3 mN m^−1^ is determined using contact angle measurements (Advance 1.6.1.0 of Krüss GmbH, Hamburg, Germany) of droplets of diiodo-methane and water on a prepared PE-HD surface (polished) with air as the surrounding phase. Its disperse fraction is 33.4 ± 0.8 mN m^−1^ and the polar fraction is 0.8 ± 0.5 mN m^−1^. This surface free energy almost matches to the values for 2 wt% Arkopal N100 solution and PFL-FR 2323. Due to the large polar contribution to the solubility parameter, water is almost insoluble in PE-HD, whereas PFL-FR 2323 contains soluble components such as solvent naphtha and cyclohexanone as shown in [31].

## 3. Methods

### 3.1. Static Loading of Compact Tension Specimen

**Figure 3:**
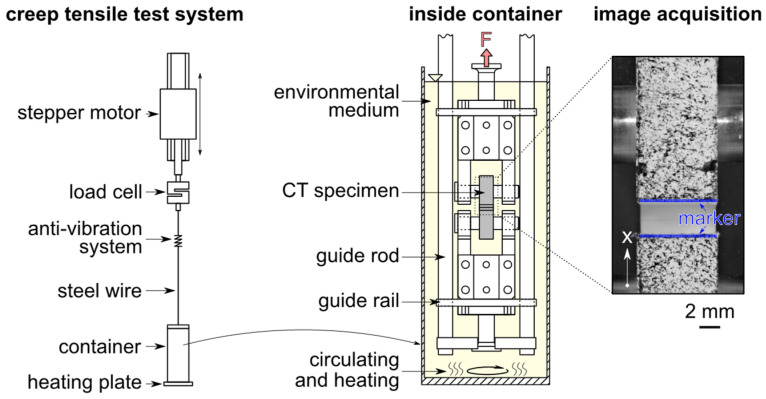
Clamping of a CT sample for static loading in a temperature-controlled environmental medium of the electronically controlled tensile creep system. Uniaxial displacement in load direction by specimen grips using guide rails along the guide rod. Image acquired with a digital camera during the experiment for optical COD(t) measurement.

Static load experimental tests are performed on an electronically controlled tensile creep test system from IPT (Institut für Prüftechnik Gerätebau, Todtenweis, Germany), Figure 3. Constant force was applied by a linear stepping motor and determined by a 500 N load cell. The specimen was immersed in a temperature controlled medium container in the clamped configuration. It was positioned precisely to determine crack opening displacement (COD(t) in Figure 2) by the optical measurement method through a quartz glass window in the medium container. The specimen clamping connection with the load cell or stepping motor was established by a steel wire. To limit the degrees of freedom of the clamping, i.e., to avoid a strong deformation by twisting of the immersed specimen under load, both the upper and lower specimen clamping was supported by a guide rail for linear displacement in the direction of load.

In the non-loaded state of this configuration, the specimen was conditioned for 1 h at the test temperature of 40 °C in the environmental medium. Subsequently, a preload of 30 N (rate 1 N s^−1^) was applied and held for 30 s before the testing load was initiated. The static tensile load of 110 N was then generated and held constant. The load of 110 N was determined in preliminary tests to be the most optimal static load considering the duration of the individual experiment and the deformation of the specimen.

For tests carried out in the solvent-based PFL-FR 2323 environment (Table 1), a state of homogeneous equilibrium with respect to the solubility of the model liquid in the PE-HD should be ensured before loading the specimen. Therefore, specimens were pre-conditioned in PFL-FR 2323 to reach the saturation concentration at 40 °C (after ca. 2000 h [15]). This ensured a constant saturation concentration without a concentration gradient across the specimen thickness at the time of loading.

### 3.2. Crack Opening Displacement Measurement

In order to determine the opening of the crack during the test, even for specimens surrounded by liquids, an optical measurement method was implemented using media containers with a quartz glass window. Based on images of the specimen’s surface taken during the test with a digital camera (DSLM Olympus E-M1 mark III) aligned with respect to the loading direction, the displacement (COD) was then determined by digital image correlation based on Matlab functions [39]. For sufficiently high magnifications, a 60 mm macro lens and extension tubes were used. The interval of image acquisitions was adjusted depending on the expected duration of the experiment and was manually controlled by Olympus Capture software. In order to enable the subsequent image analysis, a statistic color pattern was applied on the specimen prior to clamping and immersion, by speckling with a fine-pored sponge (first wetted with solvent-resistant paint) onto the specimen’s surface as shown in Figure 3.

For the analysis via digital imager correlation (DIC) with respect to COD(t), infinitesimal image sections of typically (30 × 30) pixels were defined, referred to as markers m, at the top and bottom of the notch (Figure 3 image acquisition) in a reference image n=0. Due to the inhomogeneous and statistically applied color structure on the specimen surface, these markers $m_{i,j}$ (index i and j define the marker in the specified marker matrix) are unique and enable the displacement to be followed in the next image n+1. Relative to the reference image n=0, the displacement in load direction d is determined between each image of number n by using the differences in the marker’s position $d_{m_{i,j}}^{n}=m_{i,j}^{n}-m_{i,j}^{0}$.

Clustering all markers above and all markers below the notch (Figure 3 image acquisition), an average notch opening can be calculated. For an evaluation as a function of time, the time stamp for the image n is taken from the camera generated EXIF data in the respective image file.

### 3.3. Microscopic Imaging Techniques for Fracture Surfaces

A light microscope (LM) (AxioCam ICc 3 on a Stemi 2000-C microscope, Carl Zeiss AG, Oberkochen, Germany) was used for fracture surface analysis to measure the average crack length of prematurely removed CT specimens after manual cryofracture (see also Section 4.1). A scanning electron microscope (SEM) (EVO MA10 Carl Zeiss Microscopy GmbH, Jena, Germany) with an acceleration voltage of 10 kV was primarily used to visualize the post-failure surface structures depending on the environmental medium. For this purpose, the specimens were loaded in the tensile creep device until plastic deformation was reached, the residual cross-section was then cut with a razor blade to enable exemplification of the top view in the SEM. The SEM was used to visualize the fracture surface structures in more detail with magnification levels up to 1000×. Specimens were gold sputtered in a SCD 050 sputter coater (Leica Microsystems, formerly Bal-Tec Balzers, Wetzlar, Germany) for 100 s at a current of 40 mA at room temperature reaching a layer thickness of approx. 15 nm.

## 4. Results and Discussion

### 4.1. Relation between Crack Length and Crack Opening Displacement

The determination of crack length can be measured directly by traveling microscopy along the specimen side or calculated by a compliance function through the COD. A polynomial expression describing the normalized crack length $a\;W^{-1}$ as a function of the normalized compliance of the CT specimen has been established for metallic materials and has also been proven to be valid for polymeric materials [14,40,41,42]:

(3) $$\frac{a}{W}=c_{0}-c_{1}\;U_{x}+c_{2}\;U_{x}^{2}-c_{3}U_{x}^{3}+c_{4}\;U_{x}^{4}-c_{5}\;U_{x}^{5}$$

(4) $$\mathrm{with}\;U_{x}=\frac{1}{{\left(\frac{B\cdot E\cdot \Delta COD}{F}\right)}^{\frac{1}{2}}+1}$$

Here, $c_{0}=1.001$, $c_{1}=4.6695$, $c_{2}=18.46$, $c_{3}=236.82$, $c_{4}=1214.9$ and $c_{5}=2143.6$. E is the Young’s modulus, *W* the specimen length, B the specimen thickness, and F the applied force. Since there is an initial crack opening COD_(*t*=0)_ due to the cut shown in Figure 2, the change in COD is defined as ΔCOD = COD(t) − COD_(*t*=0)_.

Polymers, and thus also PE-HD, are visco-elastic, time-dependent materials which cannot be described by a static Young’s modulus only (Equation (4)). This visco-elastic behavior requires minor modifications and changes to the analysis, as demonstrated by Rink et al. [43] as well as Andena et al. [44] and approved recently for SCG and ESC by Contino et al. [45,46,47,48] and Kamaludin et al. [49,50,51] applying LEFM. This is especially relevant in the case of indirect crack length determination using compliance analyses, representing the relationship between the monitored ΔCOD and the resulting crack length. In visco-elastic material behavior, this results in a displacement without actual crack growth. After a crack occurs, the visco-elastic-induced displacement is then also superimposed by the actual crack-induced displacement.

Therefore, the Young’s modulus is expected to depend on temperature, stress, observation time (or frequency), and strain rate as well as on plasticization by a soluble environmental liquid. All these influences are coupled with the molecular rearrangement and relaxation processes relevant to crack propagation in the semicrystalline polymer. Temperature and degree of plasticization are assumed to be constant after pre-conditioning time. However, for a creep experiment under static load, a time dependent Young’s modulus $E\left(t\right)$ has to be expected. To describe this behavior, an apparent modulus $E^{\prime }$ in Equation (4) is used [52] replacing the modulus E that is unable to describe this behavior.

**Figure 4:**
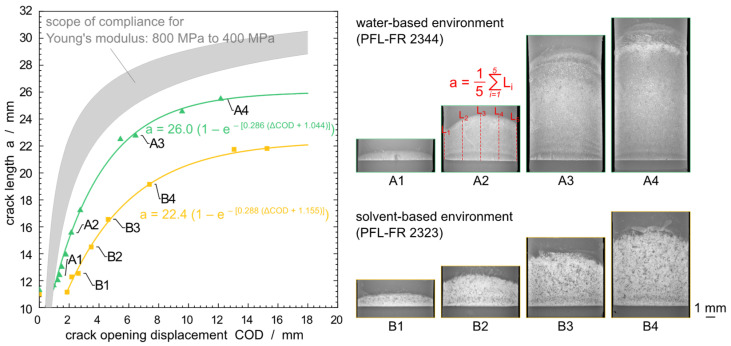
Standard polynomial compliance function, shaded gray, for Young’s modulus range of 800 MPa to 400 MPa (Young’s modulus of PE-HD at 40 °C). Experimental calibration function (exponential function fit) measured by cryogenic fracture of prematurely removed specimens in PFL-FR 2344 (green), and pre-saturated and measured in PFL-FR 2323 (yellow). Example fracture surfaces in LM from both environmental media with crack length a determination.

Since the estimation of such an apparent modulus does not seem feasible, an individual calibration of the polymer/medium system was performed. For this calibration of the relation between $a\;W^{-1}$ and the crack length, CT specimens loaded in the respective liquids were removed at specific ΔCOD values. The average crack length a was determined from LM images of the fracture surface after a cryogenic fracture, Figure 4. An average crack length a is calculated as the mean value of five readings of the crack length along the original crack front as suggested in [41]. These are measured at the edges, in the middle, and halfway between, as exemplified in Figure 4(A2).

For comparison, the standard polynomial expression describing the normalized crack length (Equations (3) and (4)) is plotted in Figure 4, shaded gray for Young’s modulus values in the range of 800 MPa to 400 MPa [53,54]. This wide modulus range is based on tensile test data reported in the literature with a strain rate of 1 mm min^−1^ to 10 mm min^−1^ at a 40 °C surrounding temperature for generic PE-HD materials. The measured average crack lengths after interrupted tests and manual cryogenic fracture as a function of the recorded ΔCOD are found to be outside this range. This discrepancy between compliance using the tensile test Young’s modulus E is not surprising, since no plasticization effects are considered and the strain rate in the creep experiments is in the order of mm h^−1^ rather than mm min^−1^ addressing tensile test requirements. In order to account for a time dependent modulus in Equation (5), we tested, as a first approximation (no relaxation time distribution, etc.), a simple Maxwell-type equation with an additional (instantaneous) elastic contribution $E_{0}$:

(5) $$E\left(t\right)=\Delta Ee^{-t/\tau_{app}}+E_{0}$$

whereby ΔE is the relaxation strength and $\tau_{app}$ is an apparent relaxation time. Since the localization of the relevant creep and relaxation processes (deformation zone at the crack tip, rearrangement within the fibrils and/or the bulk region between the crack and the fixtures for force application) and the relevant relaxation time distribution are not known, this can be only a first approach. By combining Equations (3)–(5), $a\;W^{-1}$ becomes a function of ΔCOD and time as independent variables. Although such a fit becomes unstable due to the limited number of data points ${\left(a_{i},\Delta {\mathrm{COD}}_{i},\;t_{i}\right)}^{-1}$, such an extension may be considered for further research. Fitting the experimental calibration data achieved under influence of PFL-FR 2344, the fit yields a sufficient agreement with ΔE=143.0 MPa, $\tau_{app}=15.5\;h$, and $E_{0}=269.9\;\mathrm{MPa}$. Reasonable fits are also possible within the plasticizing environment PFL-FR 2323. Equations (3) and (4), as well as Equation (5), respectively indicate alternative evaluation approaches.

However, for robustness of the fit with only one independent variable (ΔCOD) we used, in the following, an empirical calibration function to relate the ΔCOD to the measured average crack length:

(6) $$a=a_{max}\left(1-e^{-\left[\alpha \left(\Delta \mathrm{COD}+\delta \right)\right]}\right)$$

where $a_{max}$, α, and δ are empirical constants where $a_{max}$ can be related to the final crack length before ductile failure. To evaluate the measurements and to apply the fit in Figure 4, Equation (6) is used. The following values are obtained for PFL-FR 2344: $a_{max}=26.0\;\mathrm{mm}$, α=0.286, and δ=1.044 mm. The corresponding values for the solvent-based PFL-FR 2323 are $a_{max}=22.4\;\mathrm{mm}$, α=0.288, and δ=1.155 mm. Both fit functions are plotted in Figure 4. In the following calculations of this study, the calibration derived using PFL-FR 2344 as environment according to Equation (6) is used for all water-based environmental media (distilled water and 2 wt% aq. Arkopal N100), since no significant changes of material properties are expected. The plasticizing characteristic of solvents requires an additional calibration in order to determine experimental values for the studies with PFL-FR 2323.

### 4.2. Calculation of Stress Intensity Factor and Rate of Crack Propagation

The stress intensity factor $K_{I}$ of the CT specimen is calculated by Equation (1) with constant force F=110 N, specimen thickness B, as well as specimen width W (Figure 2) and normalized crack length $a\;W^{-1}$ resulting from the experimentally determined calibration function, Equation (6). The geometrical function $f\left(a\;W^{-1}\right)$ of the CT specimen is defined as follows [40]:

(7) $$f\left(\frac{a}{W}\right)=\frac{\left(2+\frac{a}{W}\right)\left[b_{0}+b_{1}\frac{a}{W}-b_{2}\;{\left(\frac{a}{W}\right)}^{2}+b_{3}\;{\left(\frac{a}{W}\right)}^{3}-b_{4}\;{\left(\frac{a}{W}\right)}^{4}\right]}{{\left(1-\frac{a}{W}\right)}^{\frac{3}{2}}}$$

with $b_{0}=0.886,\;b_{1}=4.64,\;b_{2}=13.32,\;b_{3}=14.72\;\mathrm{and}\;b_{4}=5.6$. The rate of crack propagation is determined from the crack length data as a function of time by numerical differentiation. A simple secant procedure based on the calculation of the slope of the straight line between two data points (at a time interval of 25 data points) is applied [41,55]: ${\left(\frac{da}{dt}\right)}_{\hat{a}}=\frac{\left(a_{i+25}-a_{i}\right)}{\left(t_{i+25}-t_{i}\right)}\;\mathrm{with}\;a=\left(a_{i+25}+a_{i}\right)/2$.

Since a linear relationship between crack propagation rate and stress intensity factor is often confirmed for stable crack propagation in the double logarithmic plot, the most commonly used relationship is a power law function, Equation (2).

### 4.3. Influence of Liquid Environment on Crack Propagation Rates

**Figure 5:**
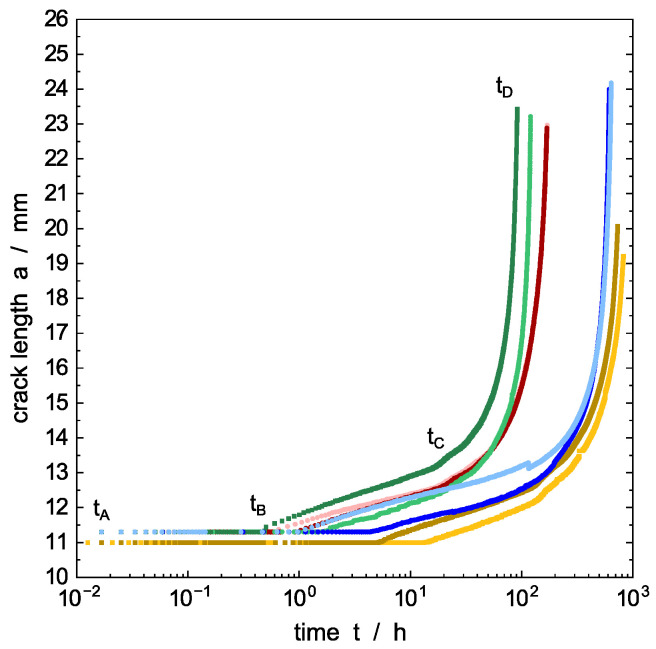
Calculated average crack length as a function of time for different environmental media at 40 °C media temperature. PFL-FR 2323 (yellow), PFL-FR 2344 (green), distilled water (blue), 2 wt% aq. Arkopal N100 (red). Different color shading represents the individual experiments performed per medium. Characteristic time used in the discussion is defined at the green curve for PFL-FR 2344.

Since all experimental parameters are kept constant and only the environmental media were varied, differences in crack propagation, and therefore the crack length progression over time in Figure 5, is attributed solely to the different physical–chemical properties of the surrounding liquids and their specific interactions with the polymer. Different colors represent the four environmental media, while different shading represents the two individual experiments performed per medium. The time to reach the maximum crack length ($t_{AD}$) by ESC in Figure 5 is defined as the time to failure ($t_{AD}=t_{f})$. The ranking, sorted with increasing time to failure, for the test liquids is: PFL-FR 2344, 2 wt% aq. Arkopal N100, distilled water, and PFL-FR 2323. Due to sorption of some components of the solvent-based PFL-FR 2323 [15] and the related plasticization of PE-HD, the crack propagation behavior for this test liquid over time cannot be ascribed exclusively to the same crack propagation mechanism as for water-based media. For PFL-FR 2323, property changes due to plasticization, rather than effects due to surfactants (ethoxylated ricinus oil and calcium alkyl benzene sulphonate in isobutanol) in the mixture, are likely to be responsible for the increased time to failure. However, other methods evaluating the ESC, such as the FNCT performed in a previous study [15], yield times to failure with the above mentioned ranking.

In the initial time interval $t_{AB}$ no crack growth could be detected, as shown in Figure 5. It should be noted that crack length starts by definition at *a*_*t*=0_ = 11 mm, Figure 2, and although a ΔCOD is detected, there is not yet any crack propagation in the initial stages. This agrees with the empirical calibration depicted in Figure 4. The time $t_{B}$ where the creep crack growth starts is referred to as initiation time. This delayed phenomenon is assumed to be connected to the microplastic flow and orientation of the amorphous phase inducing nucleation of micro-voids changing the microstructure at the base of the deformation zone (craze) upon specimen loading before the actual crack propagation [53]. It is expected that, with increasing time, crazes with elongated fibrils develop from the voids in the time interval $t_{AB}$, which, however, are not defined as a crack until $t_{B}$ is reached. Despite the difficulties in precisely determining the time when crack growth starts, $t_{AB}$ is about one order of magnitude longer for the PE-HD plasticized by PFL-FR 2323 compared to the water-based surface-active environments. $t_{AB}$ observed within distilled water does not allow a significant assignment; however, it is expected that $t_{AB}$ associates to longer times than is the case within the surface-active media.

For specimen saturated in PFL-FR 2323, the extension of the time $t_{AB}$ for preformation of the fibrillar microstructure is a result of extensive blunting, reducing the local stress concentration at the notch tip [56]. In turn, the facilitated blunting is a consequence of the reduced stiffness and strength of the polymer matrix due to plasticization [57,58]. Based on the reduced van der Waals interactions of neighboring polymer chains resulting from the incorporation of non-polar solvents, significant deformation is facilitated by the increased polymer chain mobility in the specimen, affecting not only the process zone at the crack tip, but also the entire specimen. This leads to initial crack propagation that arises comparatively later and at a larger ΔCOD than observed under the influence of non-sorptive, but surface-active, test liquids. Although PE-HD material properties are not affected by water, crack initiation is also delayed compared to the surface-active liquids. In the case of pure water as the environmental liquid, the energy for surface formation within the voids or at the crack tip is not reduced as provoked by surfactants matching the surface tension of the polymer, such as the 2 wt% aq. Arkopal N100 or PFL-FR 2344. The thus remaining resistance for crack formation due to the inferior wetting of cavities prolongs $t_{AB}$ compared to distinguished wetting characteristics of surface-active solutions. Therefore, both pre-cracking phenomena and the following crack growth are retarded.

Considering the energetic aspect of craze formation and the surface tensions of the test liquids summarized in Table 3, the relationship described by Kramer [28] emerges, that new surfaces are formed favorably if less energy is required to generate them. A relevant quantity for the energy required to form a new surface by cohesive fracture is the difference between the surface tensions of the polymer and the surrounding liquid. The surface tensions in Table 1 for PFL-FR 2344 and the 2 wt% aq. Arkopal N100 solution of 28.4 mN m^−1^ and 30.0 mN m^−1^, respectively, are close to the value of the PE-HD’s surface free energy of 34.2 ± 1.3 mN m^−1^. This implies that the micro-voids, as well as polymer-liquid interface, in the craze are favored to form (see Figure 1) when the energy required to create this is reduced. This implies, as proposed already by Kramer [28], that surface formation during the craze formation process has a significant effect on the crack propagation rate by the craze–crack mechanism of ESC.

Although the surface tension of the solvent-based PFL-FR 2323 differs not significantly from that of the aqueous detergent solution with 2 wt% aq. Arkopal N100, the crack propagation rate is significantly lower. As already stated above, PFL-FR 2323 leads to significant plasticization by incorporation of solvents with a saturation concentration of 57.9 ± 0.3 mg g^−1^ [15], mainly due to the components solvent naphtha and cyclohexanone (Table 1). Changed material properties, such as a reduction of the Young’s modulus and the yield stress, are attributed to reduced intermolecular interactions by incorporation of non-polar components between the non-polar chains of polyethylene in the amorphous phase, weakening the distance-dependent interaction of the van der Waals forces between the macromolecules, increasing chain mobility, and facilitating polymer chain disentanglement by their increased mobility [57,58]. In this context, the mobility of the amorphous phase is correlated with the amount of material drawn into the fibril from the surrounding bulk material [59]. Thereby, longer fibrils experience lower local stress. The polymer chain mobility was directly correlated with failure time, hence the higher ESCR, i.e., a decelerated crack propagation rate is expected in the case of the material plasticized by the solvents.

**Figure 6:**
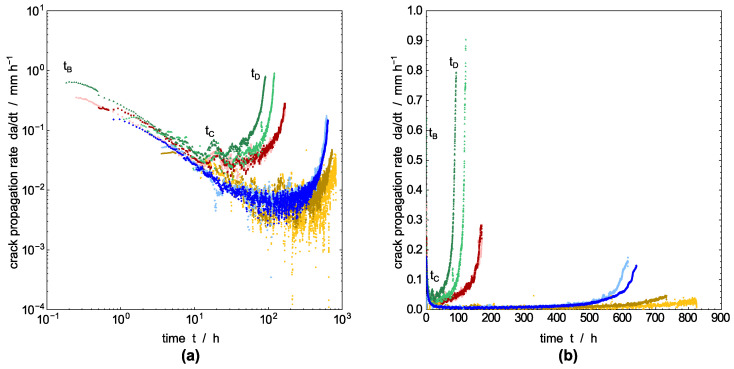
Derived crack propagation rate as a function of time for the different environmental media in (**a**) log–log basis and (**b**) linear scale. PFL-FR 2323 (yellow), PFL-FR 2344 (green), distilled water (blue), 2 wt% aq. Arkopal N100 (red).

Although the crack length progression as a function of time in Figure 5, as well as in Figure 6, for distilled water and PFL-FR 2323 look similar, the provenance driving the crack is quite different: (i) ESC in a non-plasticized material surrounded by water and (ii) ESC in a highly plasticized material influenced by PFL-FR 2323. For the latter, a competition between lower stress concentrations due to blunting by plasticization and surface tension-driven acceleration cannot be excluded. However, further research is needed to examine the effects in the initiation stage in more detail.

After $t_{B}$, a measurable crack length begins to emerge initially. Nevertheless, the crack growth rate is obviously different from the subsequent rate during $t_{CD}$. This considerably slower rate is attributed to the development of the crack from a razor blade notch [60]. Once a small crack has developed from the razor blade notch by craze–crack ($t_{BC}$), the actual crack propagation rate ($t_{CD}$) of the continuous craze–crack mechanism becomes apparent. That is, the continuing crack progresses from a pre-existing craze–crack faster than from an initial razor blade notch. This phenomenon can be minimized by fatigue cracks forming a mechanistic, incipient crack by crack growth from an introduced notch [12,55]. The transition from incipient crack growth ($t_{BC}$) to the propagation stage ($t_{CD}$) at around $t_{C}$ is more pronounced in the double logarithmic plot of the growth rate versus time in Figure 6a. A detailed analysis of the fracture surface microstructures, which mainly develop in the propagation stage ($t_{CD}$) with respect to the different liquids, will be given below in connection to the SEM images of the fracture surfaces.

The crack propagation rate, which is calculated by a numerical derivative from the values in Figure 5 as described above, is plotted in Figure 6a,b in a double logarithmic and linear scale, respectively. It shows the acceleration of the crack growth before failure during $t_{CD}$. The double logarithmic plot in Figure 6a allows a clear separation between pre-crack growth $t_{BC}$ and the actual crack growth stage $t_{CD}$. The two regions are indicated by a minimum between decreasing crack speed in the time interval $t_{BC}$ and the accelerating crack velocity in the interval $t_{CD}$ until the maximum crack length is achieved at time $t_{D}$. The decrease of crack propagation rate in the time interval $t_{BC}$ is apparently independent for all test liquids including water. As proposed by Duan and Williams [61], this behavior is related to craze formation, and an implication of flow, and the orientation of polymer chains in amorphous regions in the process zone in front of and around the crack tip [62,63]. Alternatively, Stern et al. stated in [18] that the decreasing crack speed in the beginning of the creep crack growth experiments under static load are possibly caused by a non-equilibratory size of the plastic zone introduced during the initiation time. However, the influence of pre-cracking is limited to the early state of testing [18], which is in our experiments the time interval region $t_{BC}$. The crack propagation rate for the ESC under stationary conditions is established in the time interval $t_{CD}$.

PFL-FR 2344 as a surface-active model liquid for crop protection products [15] has the strongest damaging potential for ESC, which is similar to accelerating detergents in aqueous solution [3,64,65]. The crack propagation rate for PFL-FR 2344 is significantly higher compared to the specimen immersed in distilled water, but also slightly above the crack speed achieved in the aqueous solution 2 wt% aq. Arkopal N100.

**Figure 7:**
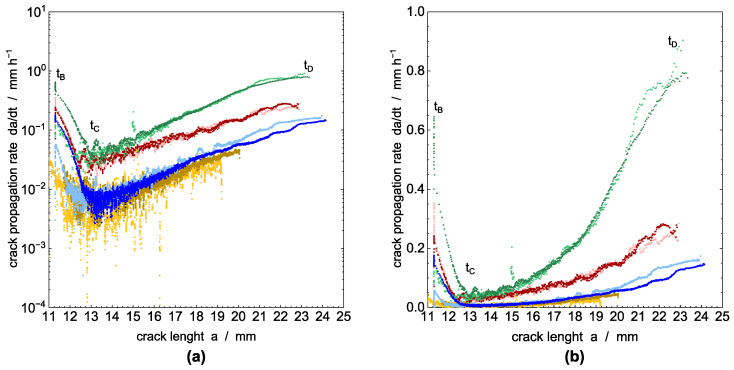
Crack propagation rate in (**a**) semi-logarithmic and (**b**) linear scale plots of static loaded CT specimens as a function of local crack length for different environmental media at 40 °C media temperature. PFL-FR 2323 (yellow), PFL-FR 2344 (green), distilled water (blue), 2 wt% aq. Arkopal N100 (red).

The significant influence of the environmental medium on crack propagation is also shown by crack speed as a function of the actual position of the crack tip, represented by the crack length in the CT specimen, Figure 7. Where Figure 7a represents logarithmic crack speed as a function of linear crack length, for Figure 7b a linear plot was chosen. The steady-state crack propagation rate representing ESC is assigned again to the time interval $t_{CD}$. Whereas the rate describing the transient crack growth ($t_{BC}$) appears to be not significantly influenced by the surrounding medium, the steady-state crack propagation rate in the interval $t_{CD}$ is clearly influenced by the environmental liquid and shows a linear increase in crack propagation rate with progressing crack length in the semi-logarithmic plot. As discussed, the most accelerated crack propagation is found for the model liquid PFL-FR 2344 for crop protection products, which is even somewhat higher than for the surface-active 2 wt% aq. Arkopal N100 solution used for accelerated testing of container materials or pipes from PE-HD.

### 4.4. Influence of Environment on the Stress Intensity Factor

**Figure 8:**
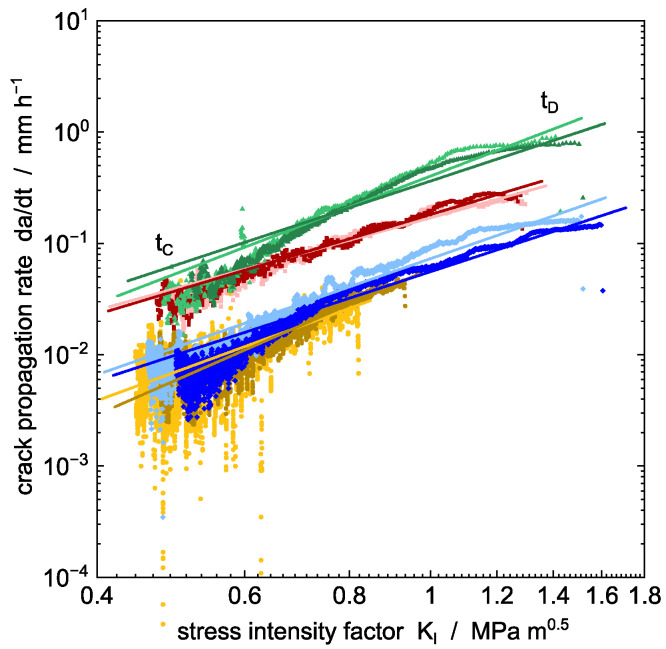
Crack propagation rate from static loading as a function of stress intensity factor. Solid lines represent a power function fit of experimental data with Equation (2). Different environmental media at a temperature of 40 °C are plotted: PFL-FR 2323 (yellow), PFL-FR 2344 (green), distilled water (blue), and 2 wt% aq. Arkopal N100 (red).

Stable crack propagation can be described by the Paris and Erdogan law [21] for dynamic cyclic fatigue experiments, where the crack propagation rate is expressed as a function of the stress intensity factor calculated by Equation (1). Application of the power law to static loads in Equation (2) is also proposed in the literature and proven [18,19,22,23]. Figure 8 shows the crack propagation rate of ESC during $t_{CD}$ for static loading as a function of stress intensity factor for all environments studied. The dependence was found to be quite general for the different surrounding liquids, independent of their tendency for sorption and plasticization or of a reduction in interface energy. The exponents m, ranging from 2.1 to 3.3, are listed in Table 4. However, Brown et al. obtained values for SCG of m = 2.6 to 4.8 [66] and m = 4.8 [67] for polyethylene materials under static load. Regarding dynamic load conditions, similar exponents of m = 3.9 are found for different PE-HD homopolymers across a variety of molecular weight distributions [42]. In both cases, the surrounding temperature was not significantly affecting the exponent.

**Figure 9:**
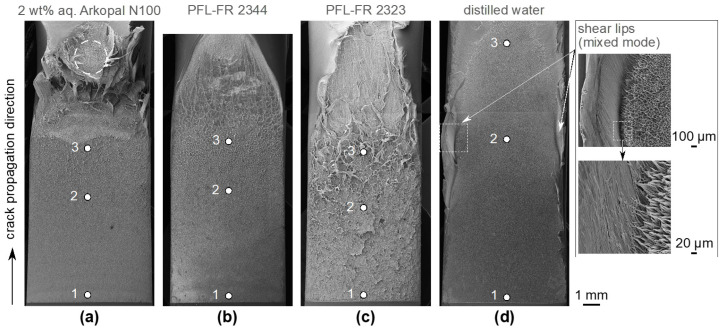
SEM images of fracture surfaces depending on the environmental medium (**a**–**d**) from the notch (bottom) to the transition from ESC to macroscopic ductile failure arresting crack propagation (top). Points 1, 2, and 3 indicate locations of detailed images in Figure 10. Formation of shear lips on the edge of the outer fracture surface along the crack propagation direction of the CT specimen when immersed in distilled water.

Reaching $K_{Ic}$ (Table 4), stable crack propagation ($t_{CD}$ in Figure 8) transitions to plastic deformation, arresting crack propagation and resulting in additional COD originating from macroscopic plastic deformation of the undamaged residual cross-section of the CT specimen (Figure 9). As given in Table 4, two specimens, pre-conditioned to reach the equilibrium saturation concentration in solvent-based PFL-FR 2323, approach the critical stress state at the crack tip by a stress intensity factor at about $K_{I}$ = 0.8 MPa m^0.5^ and 0.9 MPa m^0.5^. Due to the reduced yield stress by plasticization compared to the non-plasticized samples, chain mobility in the material is enhanced and crack propagation is retarded by macroscopic, as well as local, plastic deformation at the crack tip.

**Figure 10:**
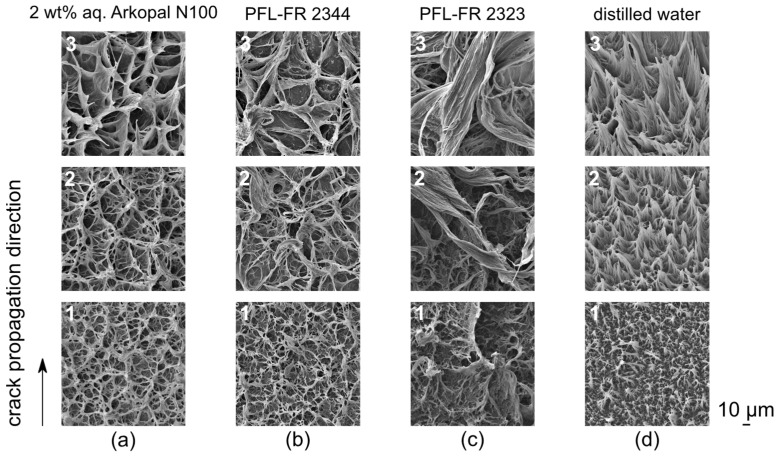
SEM detailed images of fracture surface microstructures depending on the environmental medium (**a**–**d**) and on crack length, respectively, the position 1, 2 and 3 (Figure 9) in crack growth direction on the overall fracture surface.

The relatively slow crack propagation rate due to plasticizing liquids is also due to the facilitated surface drawing in the crazing region. It is concluded that the tendency of polymer macromolecule chains to disentangle by dissipating energy into deformation is higher in the plasticized state, requiring larger deformation, i.e., larger COD, to draw the fibrils in the craze zone until failure. Facilitated surface drawing of surrounding material into the fibril results in proportionally increased elongation of fibrils until failure compared to the non-plasticized material (Figure 9 and Figure 10). In addition, it was illustrated in a previous study that the detergent components contained in PFL-FR 2323 have no distinct influence on ESC time to failure due to the significant solvent-induced plasticization of the material. As a result, with the same PE-HD material and a similar external load, i.e., at the same stress intensity factors, significantly higher crack propagation speeds are observed, caused by aggressive detergents in aqueous solution possibly accelerating damage to packaging materials for containers in critical cases.

In addition, blunting of the crack tip is also found to retard the initiation and progression of crazing [56]. While sharp crack tips induce inevitable visco-elastic deformation, a 2-phase phenomenon (bulk blunting and craze blunting) is present in the case of high stresses or considerably ductile materials, arising from the total amount of blunting [68]. Bulk blunting constitutes a large portion, significantly minimizing the stress peak at the notch tip due to its curvature. The amount of the craze-inherent blunting phase, in general crazing, is therefore diminished and dramatically retards craze formation.

### 4.5. Fracture Surface Pattern Related to Environmental Media

Crack propagation is associated with the structure of a macroscopically brittle fracture surface formed in a nominally ductile polymeric material, such as PE-HD, under low static loading conditions. The appearance of the overall characteristic pattern resulting from failed fibrillar structures of the fracture surfaces due to the craze–crack mechanism of ESC reveals the macroscopic brittle-like appearance for all test liquids of this study (Figure 9 and Figure 10). Despite all of the specific differences between test liquids, the craze–crack mechanism is a general feature related to all liquids used in this study, independent of the state of plasticization or differences in surface tension. However, there are differences between the plasticized PE-HD in solvents and the immersion in surface-active aqueous solutions, indicated by a more pronounced microstructure pattern of PE-HD in the plasticized state (Figure 9c) due to facilitated plastic flow.

Independent of the environmental medium, structures generally become larger with increasing crack length, i.e., with increasing stress intensity factor [30,69]. Accordingly, with increasing $K_{I}$ fibrils and craze structure failures have a larger average diameter (Figure 10a–d, detail 1 to 3). However, different environmental media show an impact on the formed surface structures due to failed craze fibrils. Both 2 wt% aq. Arkopal N100 and PFL-FR 2344 do not cause a significant difference in craze fibril formation, crack propagation rate and stress intensity factors being quite similar (Figure 8 and Figure 9a,b). Both also show quite similar fracture surface patterns. The more pronounced craze structure of the failed fibrils obtained (starting at the initiation of the crack) in the environmental medium PFL-FR 2323, driven by increased polymer chain mobility due to plasticization with the tendency of responding to stresses at the crack tip with more significant deformations (Figure 9c), is the result of the surface drawing into the fibril being enhanced. The three environmental media, for which wetting of the craze cavities during crack propagation can be assumed based on low surface tension (i.e., 2 wt% aq. Arkopal N100, PFL-FR 2344 and PFL-FR 2323), do not cause preferential orientation on the fracture surface. Thus, the failed fibrils are isotropically aligned in the crack propagation plane, Figure 10a–c and 1 to 3.

If the length of the stretched fibrils is a measure of its elongation at break, it is expected to depend on plasticization and the strain rate acting at the microfibrils for visco-elastic or visco-plastic materials. For the sorptive PFL-FR 2323, shear flow within the plasticized regions is expected to result in a significant extension of the elongation at break of the fibrils [57]. This may explain the elongated fibrillar structures in Figure 10c. In terms of creep, sustained stress on a plasticized material for long times may also result in such elongated fibrillar structures. On the other hand, the residual fibril structures, which form under the influence of distilled water, become larger or longer with increasing crack propagation, and in addition exhibit a preferential orientation in the direction of crack propagation, Figure 10d 1 to 3. Intuitively, an interaction with the surface tension of the surrounding medium, and the resulting wetting of the cavities and interfaces, is involved. The high surface tension of distilled water prevents or hardly wets the microfibril structures so that they remain in contact for a longer period of time without any contact with the liquid medium. Due to the prolonged existence of the fibrils, as the crack becomes more opened, the fibrils are drawn and oriented in the direction of crack propagation and purely fail because of elongation rather than medium interaction. The low crack propagation rates compared to surface active liquids also result in significantly lower strain rates locally acting at the fibrils. Consequently, sufficient time for creep deformation exists. A similar pattern of the orientation of the failed craze structures is obtained in SGC without an environmental medium [70,71].

In addition, the propagation of the crack and its transition to the failure mechanism of plastic deformation is shown in the overview SEM images, Figure 9 upper area. The plastic deformation causes a distinct necking as the ductility of PE-HD material is the property to facilitate permanent plastic deformation under shear stress before fracture, reducing the thickness of the final cross-section.

Although the focus of this paper is on the crack propagation region, it should be noted that the plastic ally deformed section develops similarly for specimens immersed in all environmental media, including in the plasticizing liquid (considering the more pronounced tendency to undergo deformation), with an exception being the deformation at the end of crack propagation in 2 wt% aq. Arkopal N100 (dotted circle in Figure 9a). Comparable observations were made for fracture surfaces from the FNCT exposed to the same test liquid [15].

An additional interesting phenomenon is the distinctive mixture of plane stress and plane strain conditions within identical experimental parameters by reason of the surrounding medium, i.e., distilled water, Figure 9d. The plane strain condition dominates towards the center of the specimen, where the thickness of the material constrains the deformation that occurs at the edges in the plane stress condition [72]. The formed shear lips along the specimen sides in the crack propagation direction are an appearance clearly related to the plane stress condition. Even though these shear lips cover only a minor proportion of the total fracture surface along the width, the condition at the crack tip is a complex mixture of plane strain and plane stress, the latter increasing with increasing crack length until the end of crack propagation. As a function of the width of a specimen (perpendicular to the direction of crack propagation), along the range of thickness from fully plane stress (i.e., thin) to fully plane strain condition (i.e., thick) there is a decrease in the stress intensity factor $K_{I}$, reaching a minimum value $K_{Ic}$ when the influences of the plane stress condition are negligible in relation to the plane strain condition and, thus, reaching a specimen-independent material parameter. Although the influence of specimen width was found to be minimal for other thermoplastics [73], the occurrence of the mixed mode condition is expected to be a characteristic dependent on the environmental medium, and therefore on the crack propagation rate. It is assumed that the intensified plane stress condition at the edges is formed in response to the slow crack propagation rates, as observed in distilled water [74].

The energy required to induce new interfaces in the crazing process is significantly higher due to the high surface tension of distilled water affecting the crack propagation behavior profoundly. In addition, crazing, being a highly intense localized flow phenomenon, absorbs more energy per volume than the more diffuse large-scale yielding [75]. In addition, the relaxation process of the stress at the crack tip is facilitated during the slow crack propagation rate. The bulk material in the plastic zone through-thickness direction is more likely to yield temporally, increasing the deformations in the zone of the plane stress condition causing shear lips. For a more reliable evaluation, further investigations should be conducted in environmental media with a relatively higher surface tension or under atmospheric conditions (air). The through-thickness evolution of the general fracture pattern with crack propagation rate increasing could not be quantitatively differentiated against the larger scaling structures of failed fibrils and crazes emerging over the entire fracture surface with increasing crack length. A more detailed physical examination of the structures, in consideration of the spatial resolved height variance of failed fibrils, could provide information about correlations of stress states since an optical evaluation by SEM does not yield reliable indications.

## 5. Conclusions

The influence of different environmental liquids (sorptive, surface-active, and inert) on ESC behavior of high-density polyethylene (PE-HD) packaging material has been demonstrated by crack propagation measurements on compact tension (CT) specimens under static load. The model liquids have been chosen with respect to common admixtures used in crop protection products. The dependence of the crack propagation rate on the stress intensity factor was described by a power law, based on the Paris–Erdogan law for cyclic testing, and was found to be quite general for the different environmental media.

Three-time regimes are observed for all test liquids (as indicated in Figure 5, Figure 6, Figure 7 and Figure 8):

(i) Microstructural changes and void nucleation: The applied load (mode I) is causing slight blunting of the tip (significant blunting for plasticized PE-HD) and, simultaneously, the formation of a process zone in front of the tip by primary orientation of polymer chains within the amorphous phase. It is expected that, with increasing time, voids elongate into cavities developing fibrils in the time interval up to *t_B_*; however, no crack is present in this time interval (*t_AB_*).
(ii) Pre-cracking: Initial fibrils of the craze zone break or disentangle starting at time *t_B_* and result in a measurable crack length. The crack propagation rate is decreasing in this time interval and a steady-state craze–crack mechanism will be approached at the end of this period (*t_BC_*). In this period, it is stated that the first notch sharpening incipient crack occurs. Additionally, a non-equilibratory size of the plastic zone introduced during pre-cracking is discussed to explain the decrease in crack velocity in this period.
(iii) Steady-state crack growth: The equilibrium crack growth stage is reached in the time interval $t_{CD}$. A stable crack propagation characteristic for the craze–crack mechanism develops. With increasing crack length, the crack propagation rate increases significantly. The crack propagation rate is related to stress intensity in this region by a power law for all media with a similar exponent. The crack propagation rate is larger for surface-active liquids compared to soluble liquids and distilled water as an inert surrounding liquid. The larger values for surface-active liquids are explained by the lower amount of energy required to form new craze interfaces. SEM images of the fracture surfaces generated in the propagation stage ($t_{CD}$) show different fibrillar structures depending on plasticization of the specimen or acceleration of crack growth by surface-active liquids.

A change in material properties due to incorporation of the solvent-based environmental medium between polymer macromolecular chains leads to higher mobility, especially in the amorphous phase, enabling the fibrils spanning the craze to draw material from the surrounding bulk, significantly increasing the elongation until failure. This surface drawing results in comparatively slow crack propagation by the craze–crack process, and not only significantly reduces the true crack propagation rate ($t_{CD}$) but also delays both void and craze formation ($t_{BC}$), as well as the formation of the incipient pre-crack as such.

From the appearance of shear lips during loading and exposure to distilled water, it is assumed that there is a relationship between the intensity of the plane stress condition and the crack propagation rate, which in turn relates energetically to the surface tension of the environmental medium. As the crack propagation rate decreases, the relaxation processes facilitate the dissipation of stresses at the crack tip by deformation in the through-thickness direction forming shear lips.

## Figures and Tables

**Table 1:** Composition of the model liquid PFL-FR 2323 for plant protection products with indication in weight percent of their amount in the mixture [35,36].

Weight %	Component
16	solvent naphtha (petroleum), heavy aromatic
16	1-methyl-2-pyrrolidone
16	1-methoxy-2-propanol
16	1,2 propanediol
16	cyclohexanone
8	ethoxylated ricinus oil
8	tap water
4	calcium alkyl benzene sulphonate in isobutanol

**Table 2:** Composition of the model liquid PFL-FR 2344 for plant protection products with indication in weight percent of their amount in the mixture [35,36].

Weight %	Component
60	sodium salt of an alkyl ether sulfate in water
25	tap water
10	1-methoxy-2-propanol
5	sodiumchloride

**Table 3:** Surface tension between the test liquid and the gas phase at 40 °C measured by the Wilhelmy plate method in mN m^−1^.

Test Liquid	Dist. Water	2 wt% aq. Arkopal N100	PFL-FR 2323	PFL-FR 2344
surface tension (at 40 °C)/mN m^−1^	68.2 ± 0.1	30.0 ± 0.2	28.4 ± 0.4	16.8 ± 0.8

**Table 4:** Fitted exponents m for the two individual experiments per medium from power law behavior and critical stress intensity factor in MPa m^0.5^ as a crucial value for transition from craze–crack propagation to macroscopic plastic deformation.

	Dist. Water	2 wt% aq. Arkopal N100	PFL-FR 2323	PFL-FR 2344
m	2.62 ± 0.02	2.08 ± 0.05	2.75 ± 0.04	2.88 ± 0.05
	2.46 ± 0.03	2.25 ± 0.03	3.30 ± 0.02	2.48 ± 0.04
$K_{Ic}$	1.60	1.29	0.93	1.52
	1.52	1.31	0.82	1.43

## Data Availability

The raw/processed data required to reproduce these findings cannot be shared at this time as the data also form part of an ongoing study.
